# Intravitreal Melphalan Chemotherapy for Vitreous Seeds in Retinoblastoma

**DOI:** 10.1155/2020/8628525

**Published:** 2020-01-24

**Authors:** Yacoub A. Yousef, Amal M. Noureldin, Iyad Sultan, Rasha Deebajah, Maysa Al-Hussaini, Munir Shawagfeh, Mustafa Mehyar, Mona Mohammad, Imad Jaradat, Ibrahim AlNawaiseh

**Affiliations:** ^1^Departments of Surgery/Ophthalmology, King Hussein Cancer Centre (KHCC), Queen Rania Al-Abdullah Street, P.O. Box 1269, Amman 11941, Jordan; ^2^Pediatrics Oncology, King Hussein Cancer Centre (KHCC), Queen Rania Al-Abdullah Street, P.O. Box 1269, Amman 11941, Jordan; ^3^Pathology, King Hussein Cancer Centre (KHCC), Queen Rania Al-Abdullah Street, P.O. Box 1269, Amman 11941, Jordan; ^4^Anesthesia, King Hussein Cancer Centre (KHCC), Queen Rania Al-Abdullah Street, P.O. Box 1269, Amman 11941, Jordan; ^5^Radiation Oncology, King Hussein Cancer Centre (KHCC), Queen Rania Al-Abdullah Street, P.O. Box 1269, Amman 11941, Jordan

## Abstract

**Objective:**

To evaluate our experience with intravitreal melphalan chemotherapy as a second-line regimen for RB patients with refractory or recurrent vitreous seeds.

**Methods:**

A retrospective case series of 16 eyes from 16 patients with intraocular RB who received intravitreal melphalan chemotherapy using the antireflux injection technique. Data included demographics, stage at diagnosis, treatment modalities, side effects, eye salvage, and survival.

**Results:**

The total number of injections was 64 (median, 3 injections per eye; range, 3–8), and the median age at time of injection was 22 months (range, 9–63 months). Nine (56%) patients were males, and 13 (81%) patients had bilateral RB. Complete response was seen in 13 (81%) eyes: in 9 (100%) eyes with focal vitreous seeds and in 4 (57%) eyes with diffuse vitreous seeds (*P*=0.062). At a median follow-up of 18 months (range, 6–48 months), the eye salvage rate was 81%, local retinal toxicity confined to the site of injection was seen in 2/3 of the eyes, 2 (12%) eyes had cataract, and none of the patients had orbital recurrence and distant metastasis or was dead.

**Conclusion:**

Intravitreal melphalan is a promising modality for treatment of vitreous seeds, and the dose of 20–30 *μ*g of melphalan sounds to be safe and effective for refractory and recurrent vitreous seeds.*μ*g of melphalan sounds to be safe and effective for refractory and recurrent vitreous seeds.

## 1. Introduction

Retinoblastoma (RB) is the most common primary intraocular malignancy in infancy and childhood [1]. There are different modalities for management of RB including chemoreduction and focal consolidation therapy to avoid enucleation or external beam radiotherapy [2–5]. Successful management is usually achieved by systemic chemotherapy and focal laser therapy, but the management of vitreous seeds and subretinal seeds is an obstacle and it affects the final prognosis and eye salvage [6, 7].

Eye salvage rate varies according to the International Intraocular Retinoblastoma Classification (IIRC) [8]. It can be as high as 100% for group A eyes, 96% for group B eyes, 90% for group C eyes, and 48% for group D eyes [9]. This lower eye salvage rate for group D eyes is usually due to active vitreous seeds. Intra-arterial chemotherapy improved the eye salvage rate for group D to 70% (64% for vitreous seeds and 83% for sub retinal seeds) [10–12]. Therefore, vitreous seeds remain a challenge to be controlled by chemotherapy as vitreous is avascular, so chemotherapy cannot reach with therapeutic levels [13]. External beam radiotherapy has been the most reliable method to treat vitreous seeds with a salvage rate of 91% [14]; however, it was associated with tremendous side effects such as secondary malignancies besides having other ocular side effects [15].

Several therapeutic agents were used for RB as thiotepa [16], melphalan [17], and methotrexate [18]. Kaneko and Suzuki [17] revealed that melphalan was found to be the most efficient drug among 12 tested drugs in vitro, and it was found to be effective and structurally nontoxic to the retina when tested on rabbits [19]. Therefore, intravitreal melphalan chemotherapy emerged as a modality of treatment for active vitreous seeds in 2012 when Munier et al. [20] reported successful treatment of vitreous seeds in 87% (20/23) of eyes by following strict protocol concerning patient selection criteria that included the absence of anterior chamber invasion, posterior chamber invasion, retinal detachment, or anterior hyaloid detachment with a strict injection technique, careful selection of tumor-free injection site, and following antireflux measures [20]. Herein, we are evaluating our experience with intravitreal melphalan chemotherapy as an eye salvage modality for eyes that harbor active vitreous seeds.

## 2. Patients and Methods

This study was approved by the institutional review board and was conducted in King Hussein Cancer Center (KHCC). It is a retrospective noncomparative study including 16 eyes of 16 patients who had intraocular retinoblastoma and active vitreous seeds and were eligible for intravitreal chemotherapy (IViC) injection as they were eligible according to the following criteria [21, 22]:

1. Absence of anterior or posterior chamber invasion
2. Absence of retinal detachment or anterior hyaloid detachment
3. Absence of tumor or vitreous seeds at entry site of injection and careful selection of tumor free injection site
4. To be performed by an experienced eye surgeon

Also, we have excluded all IIRC group E eyes and eyes with diffuse vitreous seeds involving all quadrants of the retina.

Examination under anesthesia using an indirect ophthalmoscope and fundus photography using a Retcam II (Clarity Medical Systems, Pleasanton, California) were performed for all patients before starting treatment and in each follow-up during and after the end of treatment with proper evaluation of location, extent, nature, and response of vitreous seeds. Consent was signed by parents before initiating treatment. The procedures were performed using a sterile technique in the operation room. Vitreous seeds nature was defined as focal seeds if limited to one quadrant of the globe and as diffuse seeds if extensive seeds involve more than one quadrant.

**Figure 1:**
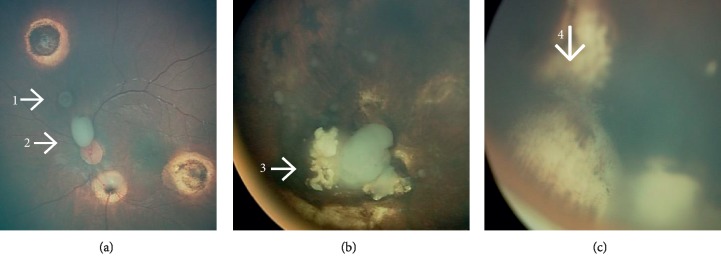
Types of vitreous seeds in retinoblastoma patients. This eye (a) harboured 2 different types of vitreous seeds at the same time: (A-1) sphere vitreous seed (type II) and (A-2) cloud vitreous seed (type III). (b) Cloud vitreous seeds (type III) and (c) dust vitreous seeds (type I).

**Figure 2:**
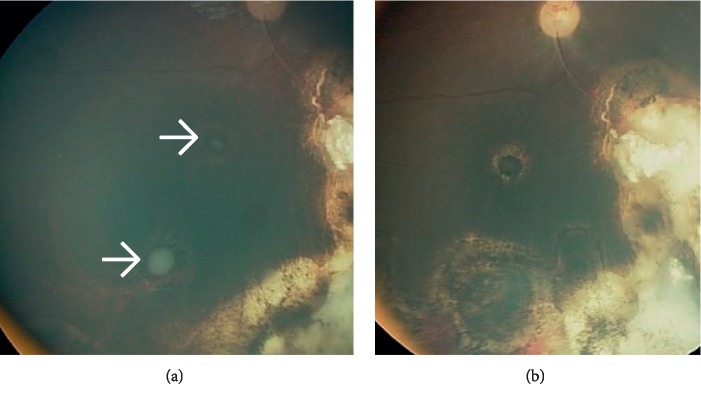
Regression of vitreous seeding: (a) cloud vitreous seeds that totally diasapperaed after 4 injections of intravitreal melphalan cyhemotherapy. (b) An example of type 0 regression pattern where vitreous seeds completely disappeared.

Distance of tumor cells in the vitreous from the retinal surface was classified into either less than 3 mm from the retinal surface or more than 3 mm. Pattern of seeds was classified into Type I dust, Type II sphere, and Type III clouds (Figure 1) [23]. Also, assessment of seeds response to treatment was classified into Type 0 (complete disappearance of seeds), Type I (calcific seeds), and Type II (amorphous seeds) (Figure 2).

### 2.1. Surgical Technique

Anterior chamber paracentesis and withdrawal of a 0.1 ml from the aqueous humor was performed before injection, and this fluid was sent for cytopathology. The site of injection was confirmed to be clear of tumor cells or retinal detachment by UBM before the injection.

A 30 gauge insulin syringe was used to inject the calculated dose. All patients received intravitreal melphalan (20–30 *μ*g according to the age) by transconjunctival pars plana route 2.5–3.5 mm from limbus towards the vitreous cavity and directed away from the lens. Two techniques were applied for prevention of passive peroperative tumor spread: the antireflux technique by inducing a transient hypotony created by withdrawal of 0.1 ml from the aqueous through the anterior chamber paracentesis and sterilization of the needle track from possible tumor cells by immediate application of triple freeze thaw cryotherapy at the site of injection. The eye was shacked cautiously after the injection to distribute the drug within the vitreous cavity, and the injection was repeated with a maximum of 8 injections at an interval of 1–2 weeks.

### 2.2. Drug Preparation

Melphalan hydrochloride (alkylating agent) is available as a 50 mg lyophilized powder that is reconstituted with preservative-free 0.9% sodium chloride solution in a sterile chamber. Initially, 10 ml of 0.9% preservative-free normal saline is added to achieve a concentration of 5 mg/ml and vigorously shaken until a clear solution is obtained. Furthermore, 1 ml of melphalan is injected into an evacuated sterile vial to which 24 ml 0.9% sodium chloride is added to yield a solution of 0.2 mg/ml (200 *μ*g/ml). The reconstituted drug (0.3 ml) is then transferred to a 1 ml luer lock syringe through a 5 *μ* filter. Dosage is adjusted accordingly (20 *μ*g/0.10 ml, 25 *μ*g/0.125 ml, and 30 *μ*g/0.15 ml) [24]. The requested dose was dependent on patients' age as follows: 0–12 months: 20 microgram, 1–3 years: 25 microgram, and 3 years: 30 microgram.

During management process: concomitant focal treatment (cryotherapy and\or transpupillary thermotherapy) was given to control the source of the seeds.

Initial response was evaluated after 1-2 weeks from first injection of initiating treatment. Success and complete response were defined as complete regression of vitreous seeds with no recurrence after the last injection and avoidance of enucleation or external beam radiotherapy (EBRT), while partial response was defined as partial, temporary regression of seeds after the last injection, and failure was defined as recurrence of seeds or growth of new seeds. Two-tailed Fisher's exact test was used to calculate *P* value.

## 3. Results

Between 2014 and 2018, 16 eyes of 16 patients with intraocular retinoblastoma who had active vitreous seeds received intravitreal melphalan chemotherapy as salvage therapy. The total number of intravitreal melphalan injections was 64 injections, and the mean number of injections per eye was 4 injections (median 3 injections, range 3–8 per eye). The dose of injected melphalan was 20–30 *μ*g.

### 3.1. Demographics and Clinical Features

There were 9 (56%) males and 7 (44%) females. Thirteen (81%) patients had bilateral disease, 3 (19%) patients had unilateral disease, and none was familial. Seven (44%) eyes had primary seeds that were resistant to the primary treatment, and 9 (56%) eyes had recurrent seeds in which 2 (12%) of them had previous radioactive plaque insertion 6 months prior to injections. The median age at time of diagnosis was 12 months (mean, 16; range, 5–48 months). According to the International Intraocular Retinoblastoma Classification (IIRC) [8], 4 (25%) eyes were group C and 12 (75%) eyes were group D. The injection was in the right eye in 8 (50%) cases and in the left eye in 8 (50%) cases. The main tumor was in the macula in 8 (50%) cases and extamacular in 8 (50%) cases. At time of melphalan injection, the other eye was enucleated in 10 (62%) patients, normal in 3 (19%) patients, and salvaged with inactive tumor in 3 (19%) eyes (Table 1).

The vitreous seeds were type I (dust) in 4 (25%) eyes, type II (sphere) in 1 (6%) eye, type III (clouds) in 9 (56%) eyes, mixture of type I and II in one (6%) eye, and mixture of type II with III in one (6%) eye. The vitreous seeds were diffuse in 7 (44%) eyes and confined to one quadrant in 9 (56%) eyes. The distance of vitreous seeds from the retina was less than 3 mm in 3 (19%) eyes and more than 3 mm in 13 (81%) eyes. Nine (56%) eyes had concomitant active subretinal seeds (Table 2).

### 3.2. Previous Treatments

Before intravitreal chemotherapy (IViC), all patients in this series had received 6–8 cycles of systemic intravenous chemotherapy (carboplatin-vincristine-etoposide), and one patient received additional topotecan chemotherapy. All patients also received focal therapy (cryotherapy and/or transpupillary thermotherapy). Two eyes (12%) had previous radioactive plaque therapy 6 months before initiating IViC, and 4 (25%) eyes had previous subconjunctival carboplatin 3 months before initiating IViC.

### 3.3. Response to IViC

Complete response was seen in 13 (81%) patients after a median of 3 injections (range 3–8), and 3 (19%) eyes showed partial regression. The type of vitreous seeds regression varied from type 0 which included complete suppression in 8 (50%) eyes, type I calcific seeds in 5 (31%) eyes, and type II amorphous seeds in 3 (19%) eyes (Figure 2). Complete response was seen in 3 (75%) eyes of group C and in 10 (83%) eyes of group D (*P*=1.0). Five (71%) of the eyes which had primary seeds showed complete response, and 8 (89%) of the eyes with recurrent seeds showed complete response (*P*=0.58) (Table 1).

Complete response of vitreous seeds was seen in 8 (89%) of the eyes that had concomitant subretinal seeds and in 5 (71%) of the eyes that had no concomitant subretinal seeds (*P*=0.55). Complete response was seen in 2 (67%) of the eyes that had vitreous seeds < 3 mm from the retina and in 11 (85%) of the eyes that had vitreous seeds > 3 mm from the retina (*P*=0.48).

Complete response was seen in 4 (57%) of the eyes that had diffuse vitreous seeds and in 9 (100%) of the eyes that had focal vitreous seeds (*P*=0.062). The median number of injections needed to get complete response was 8 injections for type II (sphere) vitreous seeds, 5 injections for mixed vitreous seeds, and 4 and 3 injections for cloud and dust seeds, respectively.

### 3.4. Management Outcome

The median follow-up after IViC was 18 months (range, 3–48 months). Recurrent or resistant vitreous seeds were seen in 3 (19%) eyes after median follow-up of 6 months (range, 3–9 months); two of them had recurrent massive vitreous seeds (3 and 6 months after last injection) and ended with enucleation. One eye had recurrence of subretinal and vitreous seeds 9 months after last injection, and the patient received further 3 cycles of systemic chemotherapy for which the tumor was resistant and external beam radiotherapy was mandatory. Retinal toxicity was detected as localized confined retinal pigmentary changes at the site of injection in 10 (62%) eyes. No eye had endophthalmitis or extraocular tumor spread. Cataract was seen in 2 (12%) eyes in which one patient had previous iodine radioactive plaque therapy, and he underwent cataract extraction surgery with intraocular lens insertion and is still stable with no recurrence 3 years after the surgery. No single patient had orbital recurrence, distant metastasis, or was dead at the last date of follow-up. The eyes that had extramacular tumor retained a median visual acuity 0.5 (range 0.2–0.8).

## 4. Discussion

Our series showed that intravitreal melphalan achieved 81% eye salvage rates in eyes that harbored active vitreous seeds and were planned for enucleation. Melphalan was chosen to be the injected chemotherapeutic agent based on studies that showed melphalan to be the most efficient among the other chemotherapeutic agents tested, showing less toxicity when used with a specific dose [25].

Safety of intraocular injection was highly questionable for decades because of the expected high risk of extraocular extension and metastasis of tumor cells in eyes with active RB. Therefore, we have followed restricted criteria in case selection, and we followed strict injection protocol to avoid extraocular tumor spread in order to guarantee safety of the procedure (that include injection in a quadrant free of tumors, decrease intraocular pressure, and cryotherapy at the site of injection), and as an outcome of that and after median 18 months of follow-up, no single patient had extraocular spread or distant metastasis. These results are comparable with those given by Munier et al. [20], who was the first to describe this approach in treating 23 eyes. In his series, no single case of metastasis was reported after a median follow-up of 22 months. Similarly, Ghassemi and Shields [26], Shields et al. [27], and Ji et al. [28], whose reports had similar eligibility criteria and injection technique, reported no single case of orbital tumor extension or metastasis in the injected 12 eyes (follow-up range, 8–66 month), 11 eyes (median follow-up, 9 months), and 19 eyes (median follow-up, 27 months), respectively. On the other hand, Kaneko and Suzuki [17] who had no well-defined selection criteria and did not follow antireflux measures reported postoperative orbital tumor recurrence in 0.4% of cases and distant metastasis in the form of intracranial invasion in 4.4% patients which is higher than our rate of metastasis. This highlights the importance of following strict criteria to our protocol to avoid any risk.

The eye salvage rate in our series was 81%, which is similar to that reported by Munier et al. [20], Ghassemi and Shields [26], Shields et al. [27], and Ji et al. [28] who reported the eye salvage rate in 87% (20/23 eyes, dose = 20–30 *μ*g), 83% (10/12 eyes, dose = 8–50 *μ*g), 100% (11/11 eyes, dose = 20–30 *μ*g), and 84% (16/19 eyes, dose = 20 *μ*g), respectively. On the other hand, Kaneko and Suzuki [17] reported the eye salvage in 68% (out of 264 injected eyes) with a slight lower dose (8–20 *μ*g). Therefore, these studies suggest that a higher dose (20–30 *μ*g) is more effective in management of vitreous RB, while lower doses are less effective.

Local toxicity of intravitreal melphalan (20–30 *μ*g) in this series was limited. The known side effects of intravitreal melphalan include anterior segment complications as uveitis and posterior segment complications as retinal toxicity, endophthalmitis, optic atrophy, vitreous hemorrhage, retinal detachment, retinal tears, extrascleral tumor extension, and metastasis. None of these was seen in our series except pigmentary retinopathy at the site of injection in 62% of cases and cataract in 2 (12%) of the cases in which intravitreal injection was related to one of them and the other one had radioactive plaque therapy previously; thus, cataract development could be related to injection or previous plaque therapy. The median visual acuity outcome in our patients was 0.5. These results are consistent with those reported by Munier et al. [20] Shields et al. [27], and Ghassemi and Shields [26] who also reported no complications other than mild localized retinal toxicity. On the other hand, Ji et al. [28] reported mild vitreous hemorrhage in 2 cases (10%) and cataract in 3 cases (16%). Similarly, Rishi et al. [29] injected 11 eyes with melphalan ± topotecan and reported anterior uveitis in 1 eye, optic atrophy in 1 eye, posterior subcapsular cataract in 2 eyes, submacular hemorrhage in 1 eye, retinopathy in 3 eyes, and subretinal fibrosis in 1 eye. Notably, Kaneko and Suzuki (who used a lower dose of 8–20 *μ*g) reported retained visual acuity 0.5 or better in 27% of the eyes that had nonmacular primary tumor, which is not better than the retained visual acuity in our patients (who received higher dose; 20–30 *μ*g) which was 0.5 or better in patients who had extamacular tumor. This indicates that the dose of 20–30 *μ*g is a safe dose for the retina in addition to being effective for vitreous seeds.

Three different types of vitreous seeds were described: dust, spheres, and clouds. Francis et al. [30] reported intravitreal injection of melphalan (30 *μ*g) for 87 patients and showed difference in time of regression of vitreous seeds according to the type of seeds where dust used to respond earlier than spheres and both responded earlier than clouds. Similarly, Rishi et al. [29] described the need for more injections to control spheres and clouds compared to dust. Similarly, the number of injections used by Ji et al. [28] to control vitreous seeds was 9, 6, and 3 injections for clouds, spheres, and dusts, respectively. In our series, control of spheres and clouds (5–8 injections) mandated more number of injections than control of dust vitreous seeds (3 injections). This indicates that dusts may harbor lower number of active cells that will be directly exposed to the chemotherapeutic agent, while in cloud tumor, cells are aggregated together so the drug will not affect the cells in the core of the cloud till they become fragments, and this is why more number of injections and longer time are needed to control cloud vitreous seeds.

Different modalities of treatment were used to treat vitreous seeds before the era of intravitreal chemotherapy. Abramson et al. [10] reported a success rate of 66% in eyes with vitreous seeds using intra-arterial melphalan, which is higher than that of systemic chemotherapy but less than the success rate of intravitreal melphalan. Similarly, Berry et al. [31] reported the probability of ocular salvage in eyes with vitreous seeding by adding radiation therapy in 64% of cases. Lee et al. [32] used combination of intra-arterial and intravitreal melphalan and reported globe salvage in 87% of cases, but that combination was associated with serious vision-threatening complications like vitreous hemorrhage and retinal detachment in half of the eyes and retinal epithelium atrophy in one third of the cases. It is not known if these complications are due to the cumulative dose of melphalan that was used or due to intra-arterial technique. Therefore, IViC is more capable of disabling active vitreous seeds vitreous seeds than systemic chemotherapy, intra-arterial chemotherapy, and radiation therapy.

Our report is retrospective in nature and evaluated a heterogeneous group of previously treated patients who needed salvage intravitreal chemotherapy to treat resistant and recurrent vitreous seeds. In our analysis, we used strict criteria to select eligible cases, and eye salvage was achieved in 81% of cases, with no serious complications. Therefore, we can conclude that intravitreal melphalan for vitreous seeds in RB is a promising modality of treatment, and the dose of 20–30 *μ*g of melphalan seems to be a safe and effective dose. We also used the special antireflux technique of injection followed by cryotherapy, which is mandatory to decrease the chance of extraocular tumor spread.

## Figures and Tables

**Table 1:** Demographics and management outcome.

		Number of eyes	Complete response (%)		Failure (%)		*P* value

Gender	Female	7	4	57	3	43	0.062
	Male	9	9	100	0	0	
Laterality	Unilateral	3	3	100	0	0	1.00
	Bilateral	13	10	77	3	23	
Vitreous seeds status	Primary	7	5	71	2	28	0.58
	Recurrent	9	8	89	1	11	
IIRC	Group C	4	3	75	1	25	1.00
	Group D	12	10	83	2	17	
Associated subretinal seeds	With SRS	9	8	89	1	11	0.55
	Without SRS	7	5	71	2	28	
Tumor location	Macular	8	5	62	3	37	0.2
	Extramacular	8	8	100	0	0	

**Table 2:** Tumor characteristics and management outcome.

				Complete response (%)		Failure (%)		*P* value

Type of vitreous seeds		Type I dust	4	2	50	2	50	0.13
		Type II sphere	1	1	100	0	0	
		Type III clouds	9	8	89	1	11	
		Mixed	2	2	100	0	0	
Distance from the retina		<3 mm	3	2	67	1	33	0.48
		>3 mm	13	11	85	2	15	
Severity of vitreous seeds		Diffuse	7	4	57	3	43	0.062
		Focal	9	9	100	0	0	

## Data Availability

The clinical data used to support the findings of this study are included within the article.

## References

[1] Kivela T. (2009). The epidemiological challenge of the most frequent eye cancer: retinoblastoma, an issue of birth and death. *British Journal of Ophthalmology*.

[2] Chan H. S. L., Thorner P. S., Haddad G., Gallie B. L. (1995). Effect of chemotherapy on intraocular retinoblastoma. *Journal of Pediatric Hematology/Oncolog*.

[3] Ferris F. L., Chew E. Y. (1996). A new era for the treatment of retinoblastoma. *Archives of Ophthalmology*.

[4] Kingston J. E., Hungerford J. L., Madreperla S. A., Plowman P. N. (1996). Results of combined chemotherapy and radiotherapy for advanced intraocular retinoblastoma. *Archives of Ophthalmology*.

[5] Murphree A. L., Villablanca J. G., Deegan W. F. (1996). Chemotherapy plus local treatment in the management of intraocular retinoblastoma. *Archives of Ophthalmology*.

[6] Shields C. L., Honavar S. G., Meadows A. T. (2002). Chemoreduction plus focal therapy for retinoblastoma: factors predictive of need for treatment with external beam radiotherapy or enucleation 11 internet advance publication at ajo.com April 8, 2002. *American Journal of Ophthalmology*.

[7] Amemiya T., Yoshida H., Ishigooka H. (1979). Vitreous seeds in retinoblastoma, clinical significance and ultrastructure. *Albrecht von Graefes Archiv für Klinische und Experimentelle Ophthalmologie*.

[8] Murphree A., Singh A. (2005). Intraocular retinoblastoma: the case for a new group classification. *Ophthalmic Oncology, Ophthalmology Clinics of North America*.

[9] Shields C. L., Mashayekhi A., Au A. K. (2006). The international classification of retinoblastoma predicts chemoreduction success. *Ophthalmology*.

[10] Abramson D. H., Marr B. P., Dunkel I. J. (2012). Intra-arterial chemotherapy for retinoblastoma in eyes with vitreous and/or subretinal seeding: 2-year results. *British Journal of Ophthalmology*.

[11] Shields C. L., Bianciotto C. G., Jabbour P. (2011). Intra-arterial chemotherapy for retinoblastoma. *Archives of Ophthalmology*.

[12] Munier F. L., Beck-Popovic M., Balmer A., Gaillard M.-C., Bovey E., Binaghi S. (2011). Occurrence of sectoral choroidal occlusive vasculopathy and retinal arteriolar embolization after superselective ophthalmic artery chemotherapy for advanced intraocular retinoblastoma. *Retina*.

[13] Gombos D. S., Cauchi P. A., Hungerford J. L. (2006). Vitreous relapse following primary chemotherapy for retinoblastoma: is adjuvant diode laser a risk factor?. *British Journal of Ophthalmology*.

[14] Shields C. L., Ramasubramanian A., Thangappan A. (2009). Chemoreduction for group E retinoblastoma: comparison of chemoreduction alone vs chemoreductionn plus low dose external radiotherapy in 76 eyes. *Ophthalmology*.

[15] Ruth A., Kleinerman, Margaret A. (2005). Risk of new cancers after radiotherapy in long-term survivors of retinoblastoma; an extended Follow up. *Journal of Clinical Oncology*.

[16] Smith S. J., Smith B. D., Mohney B. G. (2014). Ocular side effects following intravitreal injection therapy for retinoblastoma: a systematic review. *British Journal of Ophthalmology*.

[17] Kaneko A., Suzuki S. (2003). Eye-preservation treatment of retinoblastoma with vitreous seeding. *Japanese Journal of Clinical Oncology*.

[18] Kivelä T., Eskelin S., Paloheimo M. (2011). Intravitreal methotrexate for retinoblastoma. *Ophthalmology*.

[19] Ueda M., Tanabe J., Inomata M. (1995). Study on conservative treatment of retinoblastoma—effect of intravitreal injection of Melphalan on the rabbit retina. *Nippon Ganka Gakkai Zasshi*.

[20] Munier F. L., Gaillard M.-C., Balmer A. (2012). Intravitreal chemotherapy for vitreous disease in retinoblastoma revisited: from prohibition to conditional indications. *British Journal of Ophthalmology*.

[21] Munier F. L., Soliman S., Moulin A. P., Gaillard M.-C., Balmer A., Beck-Popovic M. (2012). Profiling safety of intravitreal injections for retinoblastoma using an anti-reflux procedure and sterilisation of the needle track. *British Journal of Ophthalmology*.

[22] Moulin A. P., Gaillard M.-C., Balmer A., Munier F. L. (2012). Ultrasound biomicroscopy evaluation of anterior extension in retinoblastoma: a clinicopathological study. *British Journal of Ophthalmology*.

[23] Munier F. L. (2014). Classification and management of seeds in retinoblastoma. *Ellsworth Lecture Ghent*.

[24] Manjandavida F. P., Shields C. L. (2015). The role of intravitreal chemotherapy for retinoblastoma. *Indian Journal of Ophthalmology*.

[25] Inomata M., Kaneko A. (2004). In vitro chemosensitivity assays of retinoblastoma cells. *International Journal of Clinical Oncology*.

[26] Ghassemi F., Shields C. L. (2012). Intravitreal Melphalan for refractory or recurrent vitreous seeding from retinoblastoma. *Archives of Ophthalmology*.

[27] Shields C. L., Manjandavida F. P., Arepalli S., Kaliki S., Lally S. E., Shields J. A. (2014). Intravitreal melphalan for persistent or recurrent retinoblastoma vitreous seeds. *JAMA Ophthalmology*.

[28] Ji X., Hua P., Jing Li, Li J., Zhao J., Zhao P. (2016). Intravitreal melphalan for vitreous seeds: initial experience in China. *Journal of Ophthalmology*.

[29] Rishi P., Sharma T., Agrawal V. (2017). Complication of intravitreal chemotherapy in eyes with retinoblastoma. *Oret*.

[30] Francis J. H., Abramson D. H., Gaillard M. C. (2015). Classification of vitreous seeds in retinoblastoma and response to intravitreal melohalan. *Ophthalmology*.

[31] Berry J. L., Jubran R., Kim J. W. (2013). Long-term outcomes of Group D eyes in bilateral retinoblastoma patients treated with chemoreduction and low-dose IMRT salvage. *Pediatric Blood & Cancer*.

[32] Lee J. H., Han J. W., Hahn S. M., Lyu D. J. C. J., Kim S. J., Lee S. C. (2016). Combined intravitreal Melphalan and intravenous/intra-arterial chemotherapy for retinoblastoma with vitreous seeds. *Graefe’s Archive for Clinical and Experimental Ophthalmology*.

